# The Quarantine Law of Texas

**Published:** 1899-10

**Authors:** I. J. Jones

**Affiliations:** Secretary Quarantine Department


### Public Health Department.

I. J. JONES, M. D., SECRETARY QUARANTINE DEPARTMENT.

[Each month, under this head, will be published all orders or instructions to county physicians promulgated from the office of the State Health Officer, together with abstracts of sanitary news and miscellany, by Dr. I. J. Jones, Secretary of the State Health Department.—Ed.]

### The Quarantine Law of Texas.

CHAP. 118.—[H. B. No. 623.]—An act to regulate the establishment of quarantines in the State of Texas, and in the counties, cities and towns thereof, and to repeal all laws and parts of laws in conflict therewith.

Section 1. *Be it enacted by the Legislature of the State of Texas:* That the Governor is empowered to issue his proclamation declaring quarantine on the coast or elsewhere within this State, whenever in his judgment quarantine may become necessary, and such quarantine may continue for any length of time as, in the judgment of the Governor, the safety and security of the people may require.

Sec. 2. It shall be the duty of the Governor of the State of Texas, and he is hereby authorized and empowered to select and appoint, by and with the advice and consent of the Senate, from the most skillful physicians of the State of Texas, one physician who shall be known as health officer of the State, and [who] shall from previous and active practice be familiar with yellow fever and pledged to the importance of both quarantine and sanitation.

Sec. 3. Such health officer shall, during the time he is actively engaged in public duty, receive for his services ten dollars per day* and all necessary traveling expenses, a bill of which must be made

*Changed to $2500 a year by Twenty-fifth Legislature.—Ed.

out in detail, then approved by the Governor, on which approved account the Comptroller shall issue his warrant on the Treasurer for the amount of such approved account.

SEC. 4. Whenever the Governor has reason to believe that the State of Texas is threatened at any point or place on the coast, border or elsewhere within the State with the introduction or dissemination of yellow fever, or any other infectious and contagious disease that can and should, in the opinion of the State Health Officer, be guarded against by State quarantine, he shall, by proclamation, immediately declare said quarantine against any and all such places and direct the State Health Officer to promptly establish and enforce the restrictions and conditions imposed and indicated by said quarantine proclamation, and when from any cause the Governor cannot act, and the exigencies of the threatened danger require immediate action, the State Health Officer is empowered to declare quarantine as prescribed in this article and maintain the same until the Governor shall officially take such action as he may see proper.

SEC. 5. The laws in regard to State quarantine shall remain and be in full force and operation on the coast or elsewhere in the State as the Governor or health officer may direct and be enforced as heretofore, with such additional changes as the provisions of this act and with such additional changes in station and general management as the Governor may think proper.

SEC. 6. The law in regard to local quarantine by the inhabitants of any point or points on the coast or elsewhere in the State shall remain in full force when in conformity with this act; provided, that in all differences and disputes between any such points, contiguous or remote within this State, such differences or disputes shall be immediately by the local health authorities, if any, and if none, by the inhabitants themselves, reported to the Governor, and on the receipt of such report he shall forthwith order the State Health Officer to such points with instructions to investigate the same and report the exact condition of things, and upon investigation of such report shall issue his proclamation declaring the determination of the issue, and by said proclamation the aforesaid differences shall be governed and determined.

SEC. 7. Said health officer shall give a bond with two good and sufficient sureties in the sum of ten thousand dollars, made payable to the Governor, to be approved by him, and conditioned for the honest and impartial performance of his duties, and such health officer shall hold his position for the term of two years, subject, however, to removal at any time by the Governor whenever, in his judgment, the public good demands such removal.

SEC. 8. Whenever quarantine is declared by the Governor or by any county or corporate authorities in the State, it shall be the duty of such authorities to establish a quarantine station or stations where any person may be detained for such length of time as, in the discretion of the quarantine officers, the public safety may demand; provided, that all county and municipal quarantine shall be subordinate, subject to and regulated by such rules and regulations as may be prescribed by the Governor or State Health Officer.

SEC. 9. It shall be the duty of the State Health Officer to furnish persons detained by him with necessary shelter and subsistence (not including crews of vessels, except such as are removed by the quarantine officers from infected vessels), and to provide all other things essential for the protection and comfort of those held in quarantine; and all such expenses, authorized by the State Health Officer and approved by the Governor, shall be paid by the State.

SEC. 10. All the cost and expenses of enforcing and maintaining the general quarantine or such as are ordered by the Governor or State Health Officer shall be paid out of the fund appropriated for quarantine purposes. All quarantine officers appointed by the Governor shall be selected and commissioned by the Governor of the State and shall be paid by the State, and all health authorities of the State or of any county or city thereof shall obey the rules and regulations prescribed by the Governor or State Health Officer. The regular officer in charge of regular established quarantine stations on the coast shall be allowed ten dollars per day* while on duty; temporary officers, or those commissioned by the Governor to guard against threatened epidemics, or those temporarily assigned to duty by the health officer of the State, under the provisions of Section 4 of this act, shall be allowed and paid not more than five dollars per day and such other pay for extra expenses actually incurred as may be deemed just by the Governor and State Health Officer. All quarantine officers, whether of towns, cities, counties or State shall be authorized to administer oaths to any person or persons suspected of violating any quarantine regulations; and any person or persons

*Changed to $5.00 per day at all stations except Galveston. Officers at coast stations are on duty from May 1st to November 1st The officer at Galveston is on duty all the year, and receives $2400 salary. The quarantine inspectors at Laredo, Eagle Pass and El Paso receive $1800 a year and are on duty all the year. Temporary inspectors (at Sabine River Bridge [S. P. R. R.] Logansport, Waskum, and Texarkana) are put on if yellow fever threatens, and are relieved from duty when the scare is over. They are paid $5.00 per day.—ED.

swearing falsely shall be punished according to the provisions of the Penal Code.

SEC. 11. Whenever, on the coast of Texas or elsewhere in this State, the authorities of any county, town or city, fail, refuse or neglect to establish quarantine as provided in the preceding article, then and in that event the Governor shall have the power, and it shall be his duty, to appoint a health officer and to prescribe such regulations for the government of the same as he may deem necessary.

SEC. 12. Any vessel arriving at any of the quarantine stations of this State, designated by the proper authorities, from any infected port or district without a clean bill of health from the proper officers from said port or district, shall be taken possession of by the health officer or other quarantine authority at the station at which said vessel arrives, and be held by the same until all fines that may have been assessed against the master of said vessel for a violation of the quarantine laws, rules and regulations, shall have been paid, or until said vessel shall have been replevied in accordance with law.

SEC. 13. The payment of the fine which may be assessed against the master of such vessel shall not operate as a release or discharge of the vessel from quarantine, but the same rules shall apply as in case of other vessels placed in quarantine.

SEC. 14. It shall be the duty of every county judge within the State of Texas, after each general election of State and county officers, or as soon thereafter as practicable, to select from the physicians of the respective counties, one of high character and recognized ability, who shall be known as "county physician." It shall be the duty of said county physician to establish, maintain and enforce local quarantine for his county whenever declared by proclamation of commissioners' court; to furnish supplies, select medical assistants, guards, and perform all other duties coincident to a reasonable, economic and consistent quarantine. The salary of county physician must be agreed to and paid by their respective counties, but the county physician shall receive no salary except when quarantine has been established and he is actually engaged in such service. County physicians shall in all quarantines establish rules in harmony and accord with the rules prescribed by the State Health Officer; shall respect and obey instructions from said officer, and make written reports to him of their official acts, whenever required to do, giving cause and history of epidemic, number of deaths and recoveries, and all other facts of statistic and scientific value.

SEC. 15. Whenever the commissioners' court of any county has reason to believe that they are threatened at any point or place within or without the county limits with the introduction or dissemination of a dangerous, contagious or infectious disease that can and shall be guarded against by quarantine, direct their county physician to declare and maintain said quarantine against any and all such dangerous diseases; to establish, maintain and supply stations or camps for those held in quarantine; to provide hospitals, tents or pest houses for those sick of contagious or infectious disease; to furnish provisions, medicine and all other things absolutely essential for the comfort of the well and the convalescence of the sick. The county physician shall keep an itemized account of all lawful expenses incurred by local quarantine, and his county shall assume and pay them as other claims against the county are paid. Chartered cities and towns are embraced within the purview of this article, and the mere fact of incorporation does not exclude them from the protection against epidemic diseases given by the commissioners' court to other parts of their respective counties. The medical officers of chartered cities and towns can perform the duties granted or commanded in their several charters, but must (if the county physician is not, as is frequently the case, the city physician, also) be amenable and obedient to rules prescribed by the State Health Officer. This article, however, must not be construed as prohibiting any incorporated town or city from declaring, maintaining and paying for a local quarantine.

SEC. 16. The quarantine or health officer at Galveston, Texas, shall give bond, with two or more good and sufficient sureties, payable to the Governor, in the sum of $10,000, conditioned for the care and preservation of any steam vessel or vessels belonging to the State at his station and for the faithful performance of his duty.

SEC. 17. It is hereby made the duty of the Governor and State Health Officer, upon completion of the disinfecting warehouse at Galveston or any port on the coast of Texas, to prescribe such rules and regulations as may be necessary for the disinfection of all vessels and their cargoes and passengers arriving at said ports from any infected port or district. The object of such rules and regulations being to provide safety for the public health of the State without unnecessary restrictions upon commerce and travel.

SEC. 18. All laws and parts of laws in conflict herewith are hereby repealed, and all penal statutes heretofore enacted to punish violators of quarantine laws and rules shall remain in full force.

SEC. 19. There being no law upon the subject of quarantine adequate to the protection of public health, and the near approach of the season of the year when quarantine will have to be declared, a public necessity and an emergency exists, requiring the suspension of the constitutional rule requiring bills to be read on three several days, and that this act take effect and be in force from and after its passage, and it is so enacted.

[NOTE.—The foregoing act originated in the House and passed the same, vote not given, and passed the Senate, vote not given.]

Approved April 29, 1891.

